# Decreased Sp1 Expression Mediates Downregulation of SHIP2 in Gastric Cancer Cells

**DOI:** 10.3390/ijms18010220

**Published:** 2017-01-22

**Authors:** Yan Ye, Xue Yi Qian, Miao Miao Xiao, Yu Ling Shao, Li Mei Guo, Dong Ping Liao, Jie Da, Lin Jie Zhang, Jiegou Xu

**Affiliations:** 1 Department of Immunology, Anhui Medical University, Hefei 230032, China; 18756907022@163.com (X.Y.Q.); 18226603096@163.com (M.M.X.); 15955118431@163.com (Y.L.S.); 15755161243@163.com (L.M.G.); liaodp2009@163.com (D.P.L.); zlj33@ahmu.edu.cn (L.J.Z.); 2 Department of Oncology, The First Affiliated Hospital of Anhui Medical University, Hefei 230022, China; dajie213@163.com

**Keywords:** gastric cancer, SHIP2, Sp1

## Abstract

Past studies have shown that the Src homology 2-containing inositol 5-phosphatase 2 (SHIP2) is commonly downregulated in gastric cancer, which contributes to elevated activation of PI3K/Akt signaling, proliferation and tumorigenesis of gastric cancer cells. However, the mechanisms underlying the reduced expression of SHIP2 in gastric cancer remain unclear. While gene copy number variation analysis and exon sequencing indicated the absence of genomic alterations of *SHIP2*, bisulfite genomic sequencing (BGS) showed promoter hypomethylation of *SHIP2* in gastric cancer cells. Analysis of transcriptional activity of *SHIP2* promoter revealed Specificity protein 1 (Sp1) was responsible for the regulation of SHIP2 expression in gastric cancer cells. Furthermore, Sp1 expression, but not Sp3, was frequently downregulated in gastric cancer compared with normal gastric mucosa, which was associated with a paralleled reduction in SHIP2 levels in gastric cancer. Moreover, overexpression of Sp1 inhibited cell proliferation, induced apoptosis, suppressed cell motility and invasion in gastric cancer cells in vitro, which was, at least in part, due to transcriptional activation of *SHIP2* mediated by Sp1, thereby inactivating Akt. Collectively, these results indicate that decreased expression of transcription factor Sp1 contributes to suppression of SHIP2 in gastric cancer cells.

## 1. Introduction

Gastric cancer (GC) is the fourth most common cancer and second leading cause of cancer death worldwide [1]. Advances in treatment of GC need a comprehensive understanding of its biology and behavior. Although aberrant activation of tumor suppressor genes and oncogenes has been found to be involved in the malignant behaviors of GC, it may represent only the pathogenesis of GC, and may not be specific for GC [2,3]. Thus, the role and molecular mechanisms of genetic and epigenetic changes in GC development and progression remain unclear. Our previous studies suggested the Src homology 2-containing inositol 5-phosphatase 2 (SHIP2) is commonly downregulated in gastric cancer, which contributes to elevated activation of PI3K/Akt signaling, proliferation and tumorigenesis of gastric cancer cells [4].

SHIP2, encoded by the gene *INPPL1* (inositol polyphosphate phosphatase-like 1) on human chromosome 11q13, is a member of the phosphoinositide 5-phosphatase family, which possess the common 5-phosphatase catalytic domain that can hydrolyzes the 5-position of phosphatidylinositol(3,4,5)trisphosphate (PI(3,4,5)P3) to produce phosphatidylinositol(3,4)bisphosphate (PI(3,4)P2), thereby negatively regulating the PI3K/Akt pathway [5,6]. Additionally, SHIP2 contains other protein-protein interacting domains such as an N-terminal SH2 domain, a C-terminal proline-rich domain, and a unique sterile alpha motif (SAM) domain, which affect a variety of biology behaviors, including cell adhesion, migration, invasion and receptor internalization [7,8]. These multiple-functional domains may account for the pro- or anti-tumorigenic effect of SHIP2 in different cancer cells. Although high SHIP2 expression has been found in breast cancer, hepatocellular cancer, non-small cell lung cancer, and colorectal cancer, and correlates with poor survival of patients [9,10,11,12], the reduced expression of SHIP2 and its tumor suppressive role in GC has also been reported [4]. Moreover, in squamous cell carcinoma epithelial cells, miRNA-205 targets SHIP2, thereby enhancing Akt phosphorylation and inhibiting apoptosis [13]. Also, in glioblastoma cells, overexpression of SHIP2 inhibits Akt activation and leads to cell cycle arrest [14]. Therefore, these reports, together with our findings, suggest that the pro- or anti-tumorigenic effect of SHIP2 largely depends on cell context.

However, the mechanisms underlying the abnormal expression of SHIP2 in tumors remain poorly understood. Recent studies have reported that *INPPL1* mutations were a cause of Opsismodysplasia, while increased miRNA-205 suppressed SHIP2 expression in squamous cell epithelium [13,15]. In addition, in HL60 cells and differentiated human subcutaneous white adipocytes Sp-family of transcription factors controlled human SHIP2 gene expression [16]. Accumulating evidence demonstrates that inactivation of tumor suppressor genes by promoter hypermethylation is probably an early event in gastric carcinogenesis and contributes to GC development [17]. Overall, it has been suggested that SHIP2 expression may be mediated by genetic, epigenetic, and transcriptional regulation.

Specificity protein 1 (Sp1) is a member of the Sp transcription factor family containing C_2_H_2_-type zinc fingers, and other members include Sp2, Sp3, and Sp4. They play important roles in regulation of cell survival, growth, and tumor development and progression through binding to GC-rich sequences of many cellular and viral genes [18,19]. Different from Sp2 and Sp4, Sp1 and Sp3 have the same consensus-binding sites and express ubiquitously, indicating they can act as positive or negative regulators of gene expression [20,21]. Several studies have shown that enhanced expression of Sp1 is correlated with angiogenic potential and poor prognosis in GC, whereas low expression of Sp1 has been reported to be involved in cancer progression and metastasis and may lead to poor prognosis in intestinal-type GC [22,23,24].

In the current study, we first investigated the mechanisms of decreased expression of SHIP2 in GC cells in term of genetic and epigenetic regulation. We found that genetic and epigenetic alternations are not responsible for downregulation of SHIP2 in GC cells, while transcriptional suppression of SHIP2 mediated by downregulation of the transcription factor Sp1 appears to be a commonly involved mechanism, suggesting that reduced expression of SHIP2 and Sp1 may directly contribute to GC development and progression.

## 2. Results

### 2.1. SHIP2 Is Regulated by Transcription Factor Sp1

**Figure 1:**
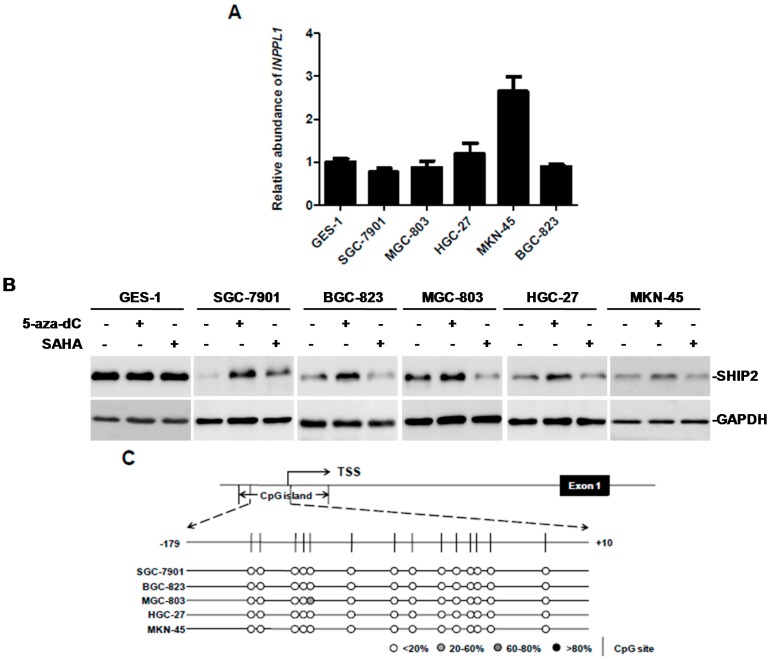

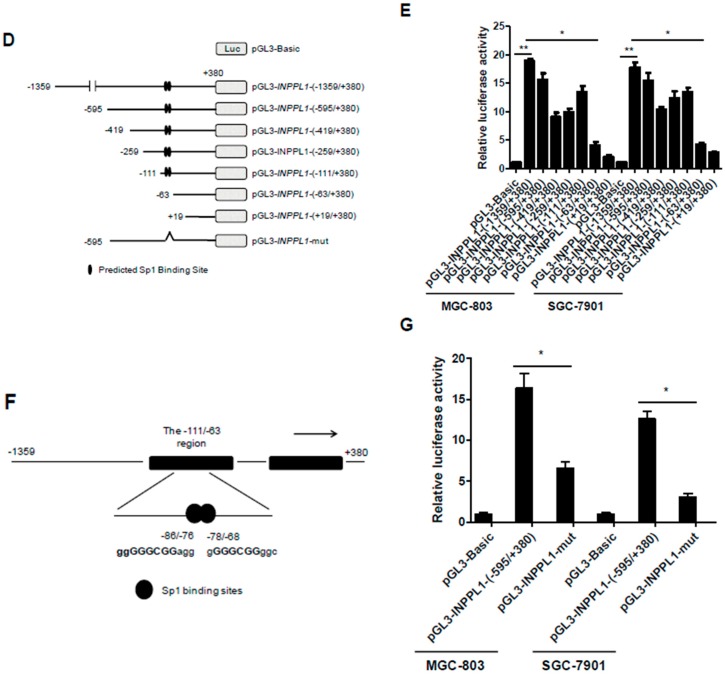
SHIP2 is regulated by transcription factor Sp1 in GC cells. (**A**) Copy number variation of *INPPL1* in the normal gastric mucosa epithelial cells GES-1 and a panel of GC cells as quantitated by qPCR analysis of genomic DNA. (*n* = 3, mean ± SEM); (**B**) whole cell lysates from GES-1 and a panel of GC cells treated with 5-aza-dC (10 μM) for 96 h or SAHA (5 μM) for 24 h were subjected to Western blot analysis. Data shown are representative of 3 individual experiments; (**C**) a typical CpG island is present at the promoter region of SHIP2. Each vertical bar represents a single CpG site. The transcription start site (TSS) is indicated by a curved arrow. BGS analysis showed no/low promoter methylation in a panel of GC cells; (**D**) a schematic illustration of construction of the luciferase reporter constructs containing a series of 5′-truncted *INPPL1* promoters and the Sp1-binding site deletion mutant; (**E**) MGC-803 and SGC-7901 cells were transiently co-transfected with pRL-TK plasmid and the pGL3-basic vector, pGL3-*INPPL1*-(−1359/+380) construct and a series of 5′-deletion mutants of pGL3-*INPPL1*-(−1359/+380). Luciferase activity was determined 48 h later, and the value was normalized to that of pGL3-basic vector designated as 1 (*n* = 3, mean ± SEM, * *p* < 0.05 vs. pGL3-*INPPL1*-(−63/+380), ** *p* < 0.01); (**F**) a schematic illustration of the Sp1 binding site-enriched fragment (−86/−68) in the −111/−63 region of the *INPPL1* promoter; (**G**) MGC-803 and SGC-7901 cells were transiently co-transfected with pRL-TK plasmid and the pGL3-basic vector, pGL3-*INPPL1*-(−595/+380) construct and pGL3-*INPPL1*-mut. Luciferase activity was determined 48 h later, and the value was normalized to that of pGL3-basic vector designated as 1 (*n* = 3, mean ± SEM, * *p* < 0.05).

By use of qPCR analysis of genomic DNA in a panel of GC cell lines and the human immortalized normal gastric mucosal epithelial cell line GES-1, we found there was no significant reduction of *INPPL1* copy number in five GC cell lines; indeed, *INPPL1* copy number was actually increased in MKN-45 (Figure 1A). Moreover, exon sequencing of all the 28 exons of *INPPL1* found no mutations in HGC-27, while the other GC cell lines had different nonsense and missense mutations at exons 9, 26, and 28 (Table 1). Collectively, these results indicate that gene copy number variation and exon mutation of *INPPL1* are unlikely to account for downregulation of SHIP2 in GC cells, suggesting that mechanisms other than genomic alterations may be the main cause of suppression of SHIP2 in GC.

Promoter hypermethylation is a common mechanism of inactivation of tumor suppressor genes contributing to gastric carcinogenesis. We therefore examined whether a similar mechanism is involved in the suppression of SHIP2 in GC cells. The methylation inhibitor 5-aza-2′-deoxycytidine (5-aza-dC) triggered an increase in SHIP2, whereas the histone deacetylase (HDAC) inhibitor SAHA did not increase SHIP2, suggesting that SHIP2 may be epigenetically suppressed by DNA hypermethylation (Figure 1B). Next, the promoter methylation status of SHIP2 was evaluated by BGS analysis. Unexpectedly, as shown in Figure 1C, in the five SHIP2-silenced GC cell lines, CpG sites of SHIP2 promoter region were methylated only in one site and one clone, indicating promoter hypomethylation of *INPPL1* in GC cells.

Next, to elucidate the transcriptional regulation mechanisms involved in suppression of SHIP2 in GC cells, we sought to determine the active region of the *INPPL1* promoter by examining luciferase activity in MGC-803 and SGC-7901 cells transfected with a pGL3-*INPPL1*-(−1359/+380) construct and a series of 5′-deletion mutants of pGL3-*INPPL1*-(−1359/+380) (Figure 1D). In both cell lines, the region from −1359 to +380 was shown to have the maximum promoter activity, and the shortened −111/+380 fragment still maintained approximately 75% *INPPL1* promoter activity. When the fragment was shortened to −63/+380, its promoter activity sharply decreased to around 20%, indicating the region from −111 to −63 contained critical regulatory elements for the promoter (Figure 1E). Furthermore, we found this minimal region contains two Sp1-binding sites, as shown in Figure 1F. When the Sp1-binding consensus sequence was deleted, the pGL3-*INPPL1*-mut construct resulted in significant reduction of promoter activity (Figure 1G). These findings are in agreement with the results obtained from 5′-deletion experiments and demonstrate Sp1 can upregulate SHIP2 transcription in GC cells.

### 2.2. Decreased Sp1 Expression Parallels the Downregulation of SHIP2 in GC

**Figure 2:**
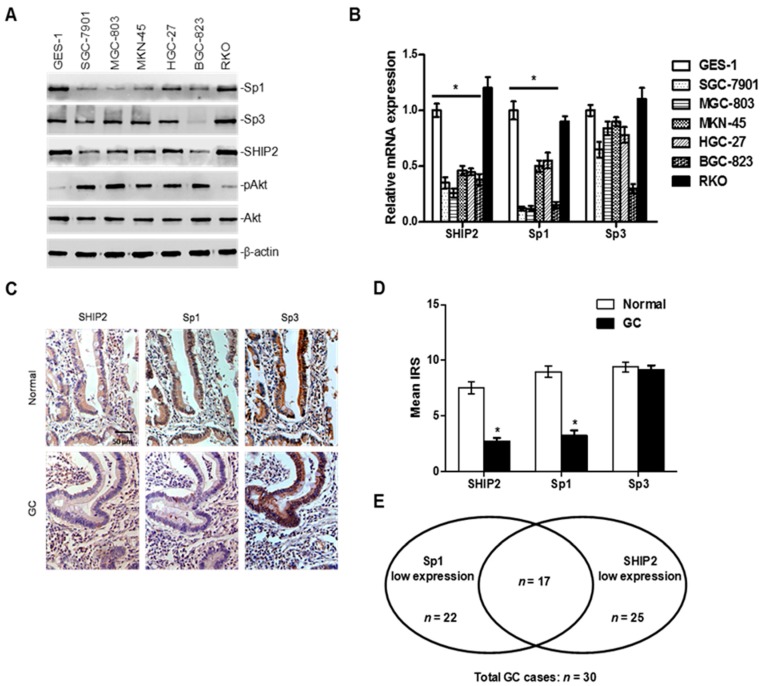
Decreased Sp1 expression parallels SHIP2 downregulation in GC cells. (**A**) Whole cell lysates from the normal gastric mucosa epithelial cells GES-1, a panel of GC cells and the human colon cancer cells RKO were subjected to Western blot analysis. Data shown are representative of 3 individual experiments; (**B**) the mRNA expression levels of SHIP2, Sp1 and Sp3 from GES-1, RKO and a panel of GC cells were analyzed by qRT-PCR. The relative abundance of target gene mRNA expression in GES-1 was arbitrarily designated as 1 (*n* = 3, mean ± SEM, * *p* < 0.01 vs. GES-1); (**C**) representative images of immunohistochemical staining of SHIP2, Sp1 and Sp3 (brown) on tissue sections from matched pairs of GC. Scale bar, 50 μm; (**D**) quantitation of SHIP2, Sp1 and Sp3 expression levels in gastric tumors (*n* = 30, mean IRS ± SEM, * *p* < 0.001 vs. normal mucosa tissues); (**E**) low expression of Sp1 and/or SHIP2 in GC tissues in vivo. Results were derived by quantitation of Sp1 and SHIP2 positive GC cells on tissue sections as shown in Figure 2C,D, and are depicted schematically.

We first determined the expression levels of SHIP2, Sp1 and Sp3 in a panel of GC cell lines by western blot and qRT-PCR. Sp1 expression, but not Sp3, was commonly reduced in GC cells compared with normal gastric mucosal epithelial cells GES-1, and this was associated with SHIP2 downregulation in GC cells (Figure 2A,B). Interestingly, the colon cancer cell line RKO expressed high levels of SHIP2 and Sp1, suggesting a different expression pattern of SHIP2 and Sp1 in gastric and colon cancer cells (Figure 2A,B). Moreover, in contrast to RKO cells, the panel of GC cell lines expressing lower levels of SHIP2 and Sp1 displayed higher levels of pAkt (Figure 2A).

IHC analysis in 30 matched pairs of GC patient specimens and tumor-adjacent normal mucosa tissues also revealed that Sp1 expression was frequently reduced in GC tissues compared with adjacent normal mucosa tissues, whereas there was no significant change in Sp3 expression between GC and normal mucosa tissues (Figure 2C,D). In particular, although there was no significant difference in distribution between Sp1 and SHIP2 expression in GC tissues (Fisher’s exact test, *p* = 0.287), approximately two-thirds (17/25) of SHIP2 low-expressing GC tissues also displayed Sp1 reduction (Figure 2E). These results suggest that downregulation of Sp1 may be a critical cause of SHIP2 suppression in GC cells.

### 2.3. Overexpression of Sp1 Inhibits Malignant Behaviors of GC Cells

**Figure 3:**
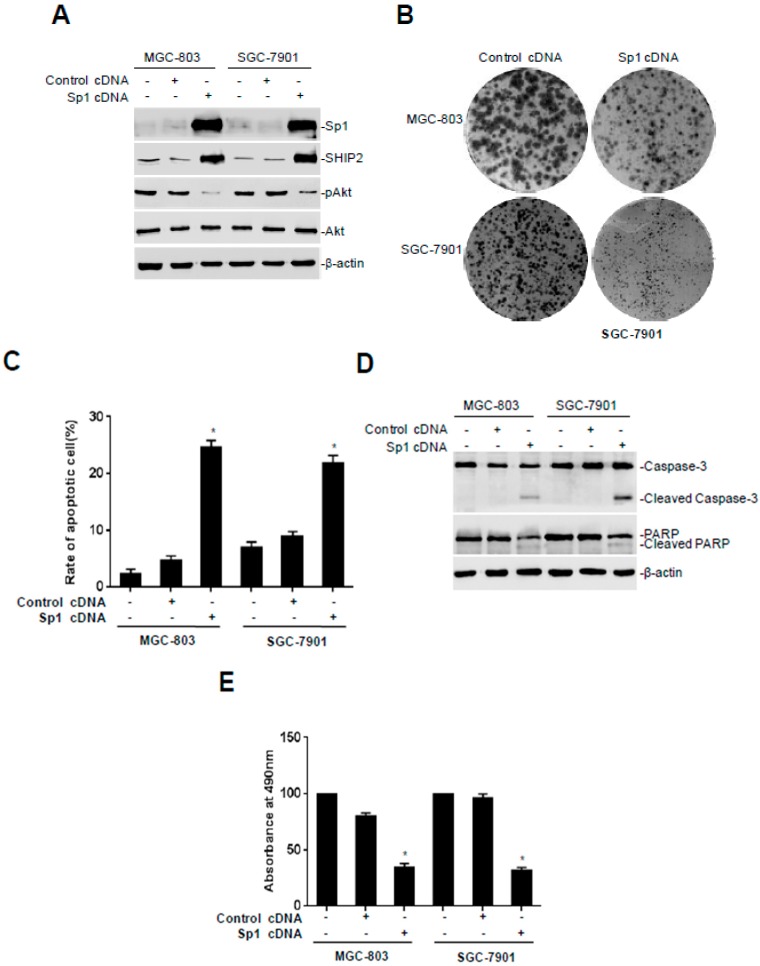

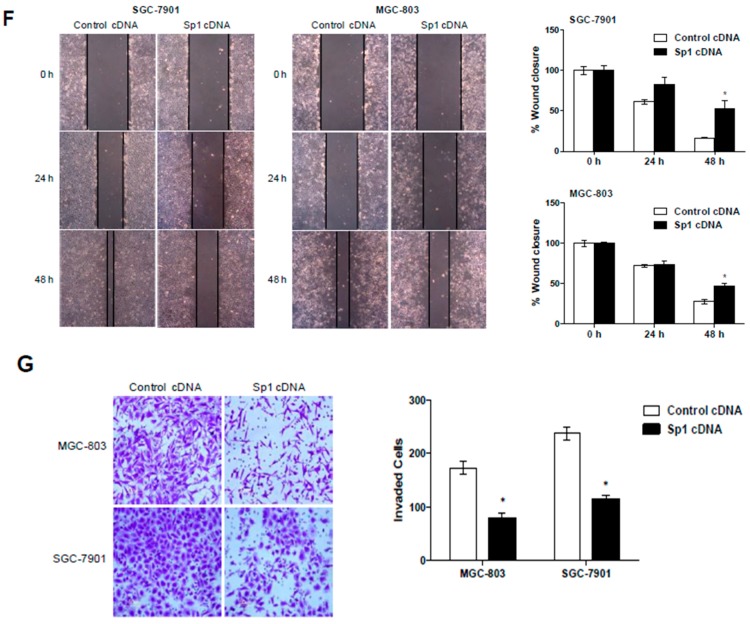
Overexpression of Sp1 inhibits malignant behavior of GC cells. SGC-7901 and MGC-803 cells were transfected with empty vector or an Sp1 construct. Data shown are representative of 3 individual experiments. (**A**) Western blot analysis of whole cell lysates; (**B**) colony formation assays of the cell growth level; (**C**) the percentage of apoptotic cells 48 h after transient transfection by measurement of sub-G1 DNA content (*n* = 3, mean ± SEM, * *p* < 0.01 vs. empty vector); (**D**) Western blot analysis of whole cell lysates for apoptosis-related proteins; (**E**) cell proliferation 72 h after transient transfection by MTS assay (*n* = 3, mean ± SEM, * *p* < 0.05 vs. empty vector); (**F**) cell migration 24 and 48 h after transient transfection by wound healing assays. Right panel shows quantitation of wound closure as shown in the left panel. The y axis represents the percentages of wound closure at 24 or 48 h after wound introduction determined using NIH Image J (*n* = 3, mean ± SEM, * *p* < 0.05 vs. transfection with empty vector for 48 h); (**G**) cell invasion 48 h after transient transfection was determined by transwell assays (*n* = 3, mean ± SEM, * *p* < 0.05 vs. empty vector).

To investigate the functional significance of Sp1 and SHIP2 downregulation in GC cells, we introduced a GFP-tagged Sp1 construct into SGC-7901 and MGC-803 cells (Figure 3A). Transient overexpression of Sp1 inhibited cell growth, as shown in Figure 3B, which was related to induction of apoptosis in a proportion of GC cells (Figure 3C,D), and inhibition of cell proliferation (Figure 3E). Moreover, SGC-7901 and MGC-803 cells transfected with Sp1 presented a slower closing in the scratch wound assay in comparison with the negative controls (Figure 3F). Also, Sp1-overexpressing cells displayed a reduced ability to invade through matrigel (Figure 3G). These results indicate that overexpression of Sp1 inhibited cell proliferation, induced apoptosis, suppressed cell motility and invasion in GC cells, which was associated with upregulation of SHIP2 and sequential inactivation of Akt (Figure 3A).

### 2.4. Overexpression of Sp1 Enhances the Transcriptional Activity of SHIP2 Promoter

**Figure 4:**
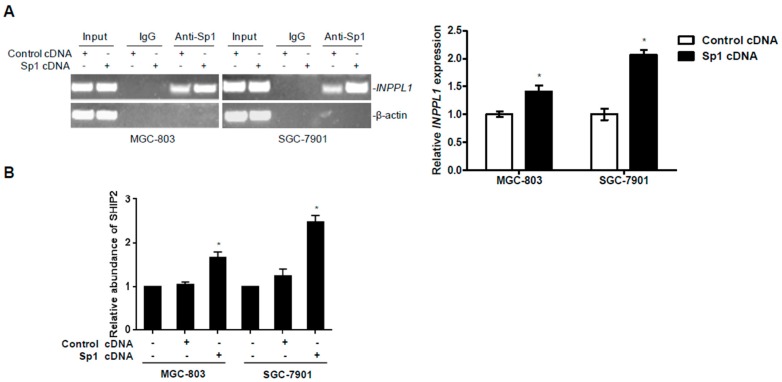
Overexpression of Sp1 enhances the transcriptional activity of SHIP2 promoter. (**A**) DNA fragments pulled down with Sp1 antibody from MGC-803 and SGC-7901 cells transfected with empty vector or an Sp1 construct were amplified by PCR. Data shown are representative of 3 individual experiments. Right column shows relative *INPPL1* expression as shown in (**A**). Levels of *INPPL1* in anti-Sp1 precipitates were normalized to those in the input. Quantitation of each band was determined using NIH Image J. The relative expression of *INPPL1* in GC cells transfected with empty vector was arbitrarily designated as 1 (*n* = 3, mean ± SEM, * *p* < 0.05 vs. empty vector); (**B**) mRNA expression levels of SHIP2 from MGC-803 and SGC-7901 cells transfected with empty vector or an Sp1 construct were analyzed by qRT-PCR. The relative abundance of SHIP2 mRNA expression in parental GC cells was arbitrarily designated as 1 (*n* = 3, mean ± SEM, * *p* < 0.05 vs. empty vector).

Previous dual luciferase reporter assays have shown that Sp1 upregulates SHIP2 transcription in GC cells (Figure 1F). To verify the transcriptional role of Sp1 in the *SHIP2* promoter, Sp1 cDNA was transfected into SGC-7901 and MGC-803 cells. Chromatin immunoprecipitation (ChIP) assays demonstrated Sp1 directly bound to the *SHIP2* promoter, and overexpression of Sp1 elevated transcriptional activation of *SHIP2* gene in the cells (Figure 4A). In addition, qPCR analysis further confirmed that SHIP2 mRNA expression was increased after Sp1 overexpression in GC cells (Figure 4B). These results indicate that binding of Sp1 to its binding sites at the −86/−68 region is required for activation of *INPPL1* transcription in GC cells.

## 3. Discussion

Aberrant expression of SHIP2 in human cancers has been found in various tumors including breast, hepatocellular, non-small cell lung, colorectal, gastric cancer cells, and aberrant SHIP2 expression is correlated with malignant potential and poor survival of patients [4,9,10,11,12]. However, the underlying mechanisms that result in abnormal expression of SHIP2 in tumors remain unknown. Our previous work identified that reduced SHIP2 expression promotes tumorigenesis and proliferation of GC via activation of the PI3K/Akt signaling [4]. Subsequently, in this report, we provide experimental evidence that decreased expression of transcription factor Sp1 contributes to suppression of SHIP2 in GC cells.

Genetic alterations such as gene copy number reduction and loss of heterozygosity (LOH) are considered to contribute to loss of tumor suppressor genes in GC. While Deng et al. have reported that the most frequently deleted chromosomal regions include 3p, 4p, 4q, 5q, 6q, 8p, 9p, 9q, 11q, 12p, 14q, 16q, 17p, 18p, 18q, 19p 21q and 22q (frequencies 7.8%–13.0%) by high resolution genomic analysis in a panel of 233 GCs and 98 matched gastric normal tissues, Arakawa et al demonstrate that 11q13.3–14.3 region (*INPPL1* maps to 11q13.4) contains copy-neutral LOH in advanced GC [25,26]. However, in our study, qPCR analysis of genomic DNA indicated no significant reduction of *INPPL1* copy number in five GC cell lines compared with GES-1, and exon sequencing did not show concordant mutations in these GC cells, which suggests genetic alternations may not be the main cause of suppression of SHIP2 in GC cells (Figure 1A and Table 1).

Epigenetic silencing of tumor-related genes due to CpG island methylation has been reported in GC, and is acknowledged as an important mechanism leading to early gastric tumorigenesis [17,27,28]. Although DNA methylation inhibitor 5-aza-dC can upregulate SHIP2 expression in GC cells, the result of BGS revealed promoter hypomethylation of *INPPL1* in GC cells (Figure 1B,C). There are two possible reasons for these contradictory results: First, DNA methylation as an epigenetic modification can control the expression of numerous genes in mammalian cells by establishing long-term gene silencing during development and cell commitment, which is essential for embryonic development. 5-aza-dC is a pan methyltransferase inhibitor that upregulates these silenced genes including housekeeping genes, tumor-related genes, and age-related genes and so on [29]. Thus, we postulate that upregulation of SHIP2 by treatment with 5-aza-dC in GC cells might be indirect. Second, the detection of methylated gene depends on the position of the CpG sites examined. In our study, BGS analysis only detected the 14 CpG sites in the central region of the CpG Island at the INPPL1 promoter region (from −179 to +10 bp) that was identified as containing key regulatory elements by transcriptional activity analysis of the *INPPL1* promoter through dual luciferase reporter assays. Therefore, these contradictory results might have been due to analysis of a limited number of CpG sites.

In the course of transcriptional activity analysis of *INPPL1* promoter, we found the minimal fragment (−111/−64 bp) required for maintaining promoter activity of *INPPL1* in GC cells and the Sp1-binding sites contained in this fragment, which is consistent with the previous report on Sp-family-mediated regulation of *INPPL1* in human adipocytes and HL60 cells [16]. However, in the case of the two GC cell lines, the transcriptional activity of the region from −419 to −112 decreased sharply and further analysis did not find any *trans*-acting factors-binding consensus sequences in this region, indicating some *cis*-acting element such as silencers may participate in the suppression of SHIP2 in GC cells.

Unlike other Sp factors, Sp1 contains a C-terminal multimerization domain which can mediate super-activation of promoters containing multiple adjacent Sp-binding sites, whereas Sp3 contains an N-terminal inhibitory domain that can repress transcription of some genes [18]. Although Sp1 and Sp3 have equal affinity for binding to the same consensus-binding motifs, their relative abundance and binding ratios influence their regulatory functions [30]. In the next, we sought the expression of Sp1 and Sp3 in GC cells to clarify the regulatory mechanism underlying SHIP2 suppression, and found that in GC cell lines and in GC tissues Sp1 expression, but not Sp3, was commonly reduced compared with normal gastric mucosal epithelial cells or matched adjacent normal tissues, which is consistent with the data in the Human Protein Atlas (available on: http://www.proteinatlas.org/ENS00000185591/cancer, http://www.proteinatlas.org/ENS00000172845/cancer). Meanwhile, paralleled expression of Sp1 and SHIP2 was observed in GC cells. Although there was no significant difference in correlated expression of Sp1 and SHIP2, the statistical sample size may be one of reasons accounting for the difference. These findings collectively suggest that decreased expression of Sp1 and SHIP2 may play a role in gastric tumorigenesis.

To better understand the role of Sp1 and SHIP2 in gastric tumorigenesis, we investigated the effect of overexpression of Sp1 on GC cell biological activity including cell growth, proliferation, migration and invasion. The results indicate Sp1 plays an important role in inhibition of cell proliferation, induction of apoptosis, suppression cell motility and invasion in GC cells in vitro. It has been reported that Sp1 is an essential transcription factor for multiple genes that are key to the regulation of physiological process and tumor development and progression [31]. Paralleled expression of Sp1 and SHIP2 and a high GC-content sequence in the proximal region of *SHIP2* promoter, the potential Sp1 binding region, indicated that SHIP2 might be a downstream gene of Sp1 in GC. The results of ChIP and dual luciferase reporter assays demonstrated that Sp1 can bind to the *SHIP2* promoter, and enhance its transcriptional activity. Nevertheless, more detailed mechanisms of SHIP2 transcription warrant further investigation.

## 4. Materials and Methods

### 4.1. Human Tissues, Cell Lines, and Cell Culture

Matched paraffin-embedded GC specimens and tumor-adjacent normal gastric mucosa tissues were collected from 30 clinically confirmed GC patients who undergone surgical resections at the First Affiliated Hospital of Anhui Medical University between 2012 and 2014. Studies using human tissues were approved by the Institutional Review Board of Anhui Medical University (No. 2015052, April 2015).

All tested human gastric cancer cell lines were purchased from Shanghai Institute of Biological Sciences, Chinese Academy of Sciences. The immortalized normal gastric mucosal epithelial cell line GES-1 was obtained from Cancer Institute & Hospital, Chinese Academy of Medical Sciences. The human colon cancer cell lines RKO was purchased from American Type Culture Collection (ATCC, Manassas, VA, USA). The cells were cultured by DMEM medium with 10% fetal bovine serum (FBS), 100 U/mL penicillin and 100 μg/mL streptomycin at 37 °C.

### 4.2. Immunohistochemistry (IHC)

Tissue sections were deparaffinized and Target Retrieval Solution (Dako, Heverlee, Belgium) was used for antigen retrieval. Tissue sections were incubated in the primary antibody, anti-SHIP2 (1:100, Abcam, Cambridge, UK), anti-Sp1 (1:100, Abcam), or anti-Sp3 (1:100, Abcam), overnight at 4 °C. Immunoreactivity was detected using the Dako Envision HRP Detection system/DAB according to the manufacturer’s instructions. IHC staining was evaluated by two independent pathologists using a previously described semi-quantitative scoring system [32]. Score for staining intensity: 0 (no staining), 1 (light brown), 2 (brown), and 3 (dark brown); for percentage: 0 (≤5%), 1 (6%–25%), 2 (26%–50%), 3 (51%–75%), and 4 (>75%). For statistical analysis, the product score (immunoreactivity score, IRS) of intensity and extent of staining was grouped into low (final score, 0 to 4) or high (final score, 5 to 12).

### 4.3. Western Blot

Thirty micrograms protein were separated by SDS-PAGE, and transferred to nitrocellulose filter membranes. After blocking with 5% skim milk for 1 h, the membranes were incubated with primary antibodies overnight at 4 °C, and then with secondary antibodies for 2 h. Primary antibodies used were as follows: monoclonal mouse anti-PARP and anti-caspase-3 antibodies (1:100, Santa Cruz Biotechnology, Inc., Santa Cruz, CA, USA); polyclonal rabbit anti-pAkt (Ser473) and anti-Akt antibodies (1:1000, Cell Signaling Technology, Beverly, MA, USA); monoclonal rabbit anti-SHIP2, monoclonal mouse anti-Sp1 and polyclonal rabbit anti-Sp3 antibodies (1:2000, Abcam); monoclonal mouse anti-GAPDH and β-actin (1:2000, Zhongshan Jinqiao, Beijing, China). The goat anti-mouse and anti-rabbit secondary antibodies are from Zhongshan Jinqiao Biotechnology (1:10,000, Zhongshan Jinqiao, Beijing, China). The images were captured and the intensity of the bands was quantitated with the Bio-Rad VersaDoc^TM^ image system (Bio-Rad, Hercules, CA, USA).

### 4.4. Plasmid Vectors and Transfection

The GV144-Sp1 construct was purchased from Genechem (Shanghai, China). Cells were transfected with 2 µg plasmid or the empty vector in Opti-MEM medium (Invitrogen, Carlsbad, CA, USA) with Lipofectamine 2000 reagent (Invitrogen) according to the manufacturer’s protocol.

### 4.5. Cell Proliferation and Colony Formation Assays

Cell proliferation was evaluated using CellTiter 96^®^ AQueous One Solution Cell Proliferation Assay (Promega, Madison, WI, USA) according to the manufacturer’s instructions as described previously [4]. For the colony formation assay, 2 × 10^3^ cells per well were plated in 6-well plates and allowed to grow for 12–14 days before fixation with methanol and staining with 0.5% crystal violet solution.

### 4.6. Apoptosis Assay

Quantitation of apoptotic cells was carried out by measurement of sub-G1 DNA content using propidium iodide (PI) on a flow cytometer as previously described [4]. In brief, 1 × 10^5^ cells per well were plated into 24-well plates and allowed to grow for 24 h followed by treatment. Cells were harvested in PI buffer, then transferred to fluorescence activated cell sorting (FACS) tubes and incubated overnight at 4 °C in the dark before analysis.

### 4.7. Wound Healing and Matrigel Invasion Assays

4 × 10^5^ cells per well were plated into 6-well plates and allowed to grow for 24 h followed by treatment. Twenty-four hours later, a scratch was introduced in each well using a pipette tip. After washing with PBS twice, serum-free medium was used for cell culture, and cells were allowed to grow for a further 24 and 48 h. Microphotographs were taken under a phase-contrast microscope.

Invasion assays were performed using a QCM^TM^ 24-Well Cell Invasion Assay Kit with 8 μm membrane (Millipore, Bedford, MA, USA). Cells were seeded at a density of 5 × 10^4^/chamber well in triplicate with serum-free medium and were allowed to invade through the matrigel for 48 h. Cells that had invaded to the lower surface of the membrane were fixed with methanol and stained with 0.5% crystal violet solution, then were photographed and counted by inverted microscope.

### 4.8. RNA Isolation and Quantitative Reverse Transcription-Polymerase Chain Reaction (qRT-PCR)

Total RNA was extracted by TRIzol Reagent (Invitrogen). Reverse transcription was performed using Moloney murine leukemia virus reverse transcriptase (Invitrogen) and random primers (Roche, Indianapolis, IN, USA). cDNA was then amplified with specific primers and Power SYBRGreen PCR Master Mix (Applied Biosystems, Foster City, CA, USA). GAPDH mRNA levels were used as an internal normalization control. The specific primers were: for SHIP2, 5′-AGCTGCCCACGCTCAAACCAA-3′ (sense), 5′-AGGTCAGGAACTGTTGGGCCGT-3′ (antisense); for Sp1, 5′-CTGCCGCTCCCAACTTAC-3′ (sense), 5′-TTGCCTCCACTTCCTCGA-3′ (antisense); for Sp3, 5′-CACTGGTCAGTTGCCAAATC-3′ (sense), 5′-GAGCTGCCACTCTTCAGGAT-3′ (antisense).

### 4.9. Chromatin Immunoprecipitation Assays

Chromatin immunoprecipitation (ChIP) assays were performed using the anti-Sp1, and ChIP assay kits (Millipore-Upstate, Temecula, CA, USA) according to manufacturer’s instructions. Briefly, cells were cross-linked using 1% formaldehyde. The bound DNA fragments were subjected to PCR reactions using the following primer pairs: INPPL1, 5′-GTTAAAGGGATTCTCCCACGG-3′ (sense) and 5′-CAAAAAAAGACCTAGCCGGTG-3′ (antisense); beta-actin (control), 5′-AGCCTTCCTTCCTGGGCATG-3′ (sense) and 5′-CCGATCCACACGGAGTACTTGC-3′ (antisense). PCR products were fractionated by gel electrophoresis and photographed.

### 4.10. Dual Luciferase Reporter Assays

The −1359/+380~−19/+380 fragments of the *INPPL1* promoter were cloned by genomic PCR using human genomic DNA as a template for the primers referred to in reference 14. The fragments were then cloned into promoter-less luciferase reporter plasmid pGL3-Basic Luciferase Vector (Promega). PCR-mediated Sp1-binding consensus deletion mutagenesis was performed using pGL3-*INPPL1*-(−595/+380) construct as a template. Cells were transiently co-transfected with pGL3-vector or pGL3-*INPPL1* or pGL3-*INPPL1*-mut construct and pRL-TK vector as an internal standard. Luciferase activity was measured using a Single-tube luminometer (Promega).

### 4.11. Exon Sequencing

Genomic DNA was isolated from GC cell lines according to manufacturer’s instructions (Promega). Samples were sequenced using *INPPL1* exon primers and data were compared to consensus *INPPL1* exon sequence.

### 4.12. Bisulfite Genomic Sequencing (BGS) Analysis

The genomic DNA isolated from GC cells was modified by bisulfite treatment using CpGenome^TM^ Fast DNA Modification Kit (Millipore) according to manufacturer’s instructions. Bisulfite-treated DNA was amplified using BGS primers, and the PCR products were subcloned into the pGEM-T Easy vector (Promega). Five randomly clones of each specimen were chosen and sequenced. The BGS primer sequences were 5′-AGTTTTTTTTTAGGTTAAAGGG-3′ (sense) and 5′-CCCATAAAAAAATCTAACCC-3′ (antisense), which generated a 189 bp fragment containing 14 CpGs at the *INPPL1* promoter region (from −179 to +10 bp).

### 4.13. qPCR Analysis of Copy Number Variations

Genomic DNA was extracted from GC cells and normal gastric mucosal epithelial cell line GES-1. DNA was quantified using a NanoDrop spectrophotometer, and quality was assessed by agarose gel electrophoresis. The specific primers used to examine copy number variations (CNVs) of *INPPL1* were: forward 1, 5′-TCCTGGCTTCGGACTGCTGA-3′; reverse 1, 5′-GACGAGGTGCGACGTTCTCG -3′; forward 2, 5′-CATTGGGAGAACTGAGAAGCG-3′; reverse 2, 5′-CCATCATTCACCCGAGGAGAT-3′. As a control, the housekeeping *HBB* was amplified and quantitated. The primers for HBB were: forward, 5′-ACACAACTGTGTTCACTAGC-3′; reverse, 5′-CAACTTCATCCACGTTCACC-3′.

### 4.14. Statistical Analysis

Fisher’s exact test was used to determine the correlation between the expression of SHIP2 and Sp1 in human gastric cancer. The two-tailed Student’s *t* tests were used to evaluate the other statistical differences. A *p*-value < 0.05 was defined as statistical significant.

## 5. Conclusions

In summary, we showed that Sp1 upregulated SHIP2 expression at the SHIP2 promoter activity, mRNA, and protein levels via directly binding to *SHIP2* promoter in GC cells. Paralleled reduction of Sp1 and SHIP2 may contribute to GC development and progression.

## Figures and Tables

**Table 1:** SHIP2 mutations in gastric cancer lines.

Cell Lines	Nucleotide Change	Status	Amino Acid Change	Location	Domain
HGC-27	N	–	N	–	–
SGC-7901	c.1134A>G	ho	p. 329Ser *	Ex9	/
	c.3395C>G	ho	p.Ala1083Gly	Ex26	Pro-rich domain
	c.4046_4061del	ho	?	Ex28	/
	c.4192G>C	ho	?	Ex28	/
MGC-803	c.1143A>G	he	p. 329Ser *	Ex9	/
	c.C3395C>G	he	p.Ala1083Gly	Ex26	Pro-rich domain
	c.4046_4061del	he	?	Ex28	/
MKN-45	c.1143A>G	ho	p. 329Ser *	Ex9	/
	c.3133C>A	he	p.Leu996Met	Ex26	Pro-rich domain

Abbreviations are as follows: del, deletion; ho: homozygote; he, heterozygote; N, no mutation; –, no; /, mutation not localized in a known protein domain; ?, unknown; *, nonsense mutation.
